# Supplementary material for: Research data sharing in the Australian national science agency: Understanding the relative importance of organisational, disciplinary and domain-specific influences
Source: PLoS One. 2020 Aug 28;15(8):e0238071. doi: 10.1371/journal.pone.0238071 (PMC7454993; doi:10.1371/journal.pone.0238071)
Supplement: Attachment — Submitted filename: Response to Reviewers PONE-D-20-07169(b).docx [file pone.0238071.s004.docx]

Dear Professor Rozylowicz,

We are grateful to you and the reviewers for your constructive feedback and the opportunity to revise our manuscript. We appreciate the time and effort that has gone into the initial review process.

Below, we have reproduced the feedback from you and the reviewers (in italics), along with an explanation of the way in which we have responded to this feedback.

## Editor’s feedback

*The title of the manuscript needs to be more focused, the paper rationale and objectives should be more clear and in line with reported results. The methodology section needs an extensive revision, as some analyses are not reported or not clear, and the sampling is not well explained to the readers. As the reviewers pointed out, data behind the analyses are not available for readers. Please include the dataset in the next version, e.g., by anonymizing the data. If not possible, please be more explicit about why this is the case.*

The revised manuscript has a new title and the Introduction has been edited to focus in on our research objectives. We have also revised the Method section, providing information about the sample and clarifying ambiguities in the methodology.

In the Results section we have added a new table which lists the survey items and provides the output from the factor analysis (see the new Table 2). We have also published the data aggregated by organisational unit, discipline group and program (along with the code used for our analyses) on CSIRO’s Data Access Portal, at <https://data.csiro.au/dap/landingpage?pid=csiro:44660>. However, we could not include all of the organisational units tested in our analyses because we only obtained informed consent to publish aggregated data, where the aggregations were based on responses from 10 or more survey participants. In consequence, there are some inconsistencies between the statistics reported in our manuscript and statistics derived from the published data.

*To enhance the reproducibility of your results, we recommend that if applicable you deposit your laboratory protocols in protocols.io, where a protocol can be assigned its own identifier (DOI) such that it can be cited independently in the future. For instructions see:* [*http://journals.plos.org/plosone/s/submission-guidelines#loc-laboratory-protocols*](http://journals.plos.org/plosone/s/submission-guidelines#loc-laboratory-protocols)

We have published the code that we used for our analyses (for the purposes of transparency) but it is not possible to reproduce our results with this code because we do not have consent from our participants to publish the de-aggregated data. The code is published in the same location as the data, on CSIRO’s Data Access Portal at: <https://data.csiro.au/dap/landingpage?pid=csiro:44660>

## Journal Requirements:

*When submitting your revision, we need you to address these additional requirements.*

*1. Please ensure that your manuscript meets PLOS ONE's style requirements, including those for file naming. The PLOS ONE style templates can be found at*

[*http://www.journals.plos.org/plosone/s/file?id=wjVg/PLOSOne_formatting_sample_main_body.pdf*](http://www.journals.plos.org/plosone/s/file?id=wjVg/PLOSOne_formatting_sample_main_body.pdf) *and* [*http://www.journals.plos.org/plosone/s/file?id=ba62/PLOSOne_formatting_sample_title_authors_affiliations.pdf*](http://www.journals.plos.org/plosone/s/file?id=ba62/PLOSOne_formatting_sample_title_authors_affiliations.pdf)

Before submitting the revised manuscript we revisited PLOS ONE’s style requirements provided at the above links. We checked that the revised manuscript reflected the style requirements but could not find specific advice regarding file names.

*2. Please include additional information regarding the survey or questionnaire used in the study and ensure that you have provided sufficient details that others could replicate the analyses. For instance, if you developed a questionnaire as part of this study and it is not under a copyright more restrictive than CC-BY, please include a copy, in both the original language and English, as Supporting Information.*

The survey items are now listed in the manuscript as part of the factor analysis output (see Table 2).

*3.  We note that you have indicated that data from this study are available upon request. PLOS only allows data to be available upon request if there are legal or ethical restrictions on sharing data publicly. For more information on unacceptable data access restrictions, please see* [*http://journals.plos.org/plosone/s/data-availability#loc-unacceptable-data-access-restrictions*](http://journals.plos.org/plosone/s/data-availability#loc-unacceptable-data-access-restrictions)*.*

*In your revised cover letter, please address the following prompts:*

*a) If there are ethical or legal restrictions on sharing a de-identified data set, please explain them in detail (e.g., data contain potentially sensitive information, data are owned by a third-party organization, etc.) and who has imposed them (e.g., an ethics committee). Please also provide contact information for a data access committee, ethics committee, or other institutional body to which data requests may be sent.*

*b) If there are no restrictions, please upload the minimal anonymized data set necessary to replicate your study findings as either Supporting Information files or to a stable, public repository and provide us with the relevant URLs, DOIs, or accession numbers. For a list of acceptable repositories, please see* [*http://journals.plos.org/plosone/s/data-availability#loc-recommended-repositories*](http://journals.plos.org/plosone/s/data-availability#loc-recommended-repositories)*.*

The survey was commissioned by the CSIRO and the data were collected from employees of the CSIRO. Since there is a higher degree of sensitivity when you are collecting data from people in a workplace that is the subject of the research, the ethics approval was granted on the condition that only aggregated data would be published. When participants provided their consent to participate in the survey, it was on the condition that “In any instances where the data are

reported (whether as a presentation or publication) the data will be aggregated to ensure that your

individual responses cannot be identified (data will not be reported for groups where there are fewer than 10 participants represented in the dataset).” In line with these conditions, we have published aggregated data for each organisational unit, disciplinary group and domain group. Since ten of the organisational units had fewer than 10 respondents, we had to exclude data from 10 of the organisational units that were included in our own analyses. In consequence, it is not possible to replicate the statistics from our organisational analyses. However, as noted earlier, it is not possible to replicate the multi-level analyses using aggregated data in any case.

*We will update your Data Availability statement on your behalf to reflect the information you provide.*

Thank you.

## Reviewer #1:

*The manuscript addresses a relevant research topic for nowadays science: data-sharing practices. Overall, the manuscript is presented in an intelligible fashion and written in standard English, while the statistical analysis was performed appropriately and rigorously. Even if the manuscript has merits, there are several aspects that need authors attention and impede the potential publication.
I would start by stressing the irony surrounding this paper: comparing the results reported by the authors and their justification referring to the availability of the data-set supporting this manuscript.*

Our difficulty sharing the data from this human research project serves well to illustrate the organisational and disciplinary impacts on data sharing. Since our research involves human participants (the first author’s field expertise is in the organisational psychology domain) the research had to go through review by a human research ethics committee. Furthermore, because the participants in the study were employees of the organisation conducting the research, we specified that only aggregated data for groups represented by ten or more respondents would be published. However, since it is difficult to achieve a good response rate to organisational surveys, not all organisational units, research disciplines and domains met this criterion. For our analyses, we chose to include units with seven or more respondents represented since this is the criterion for calculating intraclass correlations for survey measures with a 7-point Likert scale and it provided more cases for our analyses. In line with our ethics requirements, we have published the data for those organisational, disciplinary and domain groups with ten or more respondents represented but we note that these data do not allow other researchers to replicate our analyses precisely. The published data are now available via CSIRO’s Data Access Portal at: <https://data.csiro.au/dap/landingpage?pid=csiro:44660>

*#1. The title of the manuscript is too general (e.g. it should stress that it is a research conducted on an Australian governmental agency). From a certain perspective, some would argue that it could potentially mislead readers.*

We have revised the title of the manuscript so that it is more focused. Whereas the original title was “Modelling organisational, disciplinary and domain-specific sources of influence in research data sharing” the new title is:

“Research data sharing in the Australian national science agency: Understanding the relative importance of organisational, disciplinary and domain-specific influences”

*#2. The problem or the rationale of the paper is clouded by the diffuse writing. It should clearly answer "why the effort?" and also the contribution to the field.*

We have re-written the introduction section of the manuscript to highlight the importance of institutional initiatives aimed at supporting research data sharing and the need to understand the relative importance of different sources of influence (organisational, disciplinary and domain). We have also simplified the wording of the manuscript as much as possible.

*#3. The abstract contains extremely detailed information on the results (e.g. that organizational unit membership explained 10%, disciplinary membership explained 6%, and domain membership explained 4% of the variance in researchers’ intentions to share research data) and ignores for instance the limits, the contribution to the field, etc. Additionally, it refers to specific concepts that cannot be understood appropriately unless the entire content is read (e.g. contractual and regulatory inhibitors).*

The abstract has been re-written to highlight the paper’s contribution to the field. We have retained the information about the proportion of variance in intentions to share data that is explained by organisational, discipline and domain membership since delineating the relative importance of these sources of influence represents the central contribution of the study. However, we translate these statistics into the key finding, namely: “whereas previous research has tended to focus on disciplinary influences on data sharing, this study suggests that factors operating within the organisation have the most powerful influence on researchers’ data sharing practices.” We also provide examples of contractual inhibitors (“e.g., ownership or licensing arrangements”).

*#4. The theory section does not support the variables introduced into the statistical models. Recent literature on the topic is missing. I would suggest the authors to re-shape and re-frame the theory part of their paper.*

As requested, we have re-written our introduction section so that it is more concise and directly focuses on the theory and evidence for predicting organisational, disciplinary and domain influences on data sharing. We have updated the literature to include three more recent references (Kim & Yoon, 2017; Tenopir et al., 2020; Unal, Chowdhury, Kurbanoğlu, Boustany, & Walton, 2019) which provide additional support for the need to delineate disciplinary, organisational and domain-related influences on research data sharing.

*#5. The authors do not explain why academic and non-academic data are equivalent (see section "Application domain").*

We did not mean to imply that academic and non-academic data are equivalent but rather, to illustrate both formal (field of research classifications) and informal (funding organisations) ways in which a researcher’s domain can be identified. To clarify this point we have re-worded the first paragraph of the “Application domain” section in the manuscript (see page 5, line 126 up to page 6, line 131).

*#6. It is safe to delete Figure 1 - it is redundant, it does not provide any useful information to the readers.*

We have adopted this suggestion and removed Figure 1.

*#7. Background information on the respondents is missing (publication records, research impact, tenure, experience etc.). Also, it is not self-evident how representative are respondents for the Australian research community.*

We have provided more contextual information about the CSIRO and its role and importance in Australia’s research and innovation system. This information is provided in the first paragraph of the Method section (under “Sample”).

*#9. Authors should make their methodology clear. See the below questions for illustration:
- How were the employees recruited? Why CSIRO? Is it relevant for Australia?
- What is the total number of employees at CSIRO? Did the authors send emails to everybody? What is the meaning of 22%? Is the sample relevant for the entire agency? But for the entire Australia?
- Authors should explain the methodological limits for this paper's results and findings in the context of working with a convenience sample.
- Some of the disciplines are better represented in the sample. Does that impact upon the results? What is the structure of the agency's workforce on these research disciplines and application domains?
- Lines 200 - 205: How many were there? What were the criteria applied by the Information Services expert on selecting the "active research roles". Did he do a good job, given that some of the respondents reported 0 time spent on research data?
- Some of the text in the Methods sections is redundant or common knowledge, e.g. "The statistical modelling was carried out using R [29], an opensource language and environment for statistical computing."*
*- Text between lines: 298 - 305 should be moved somewhere in the above section, as it does not report any results. It is only technical procedure.*

In the revised manuscript (see final paragraph of page 8, up to page 9, line 205) we provide better information about the size, nature and importance of the CSIRO in Australia. We report the total number of employees in CSIRO and provide more detail on the procedure for recruiting employees and cleaning the dataset. We have also condensed the Method section to remove redundant information. Finally, we acknowledge the limitations of the sample in the discussion section of the manuscript.

We have not moved the text relating to the factor analysis (this is the text which originally appeared on lines 298-305) since Reviewer 2 requested that more detail be provided on the factor analysis. In the revised manuscript we have added a table (Table 2) which shows the scale items and their factor loadings.

*#10. Some parts of the text in the discussion section are not supported by the results, e.g. "The failure to observe significant effects for the other variables in the model may simple reflect low statistical power."*

We have retained this statement in the Discussion section but we justify it with further detail. The manuscript now explains that power in a multilevel analysis reflects the number of observations at the level of the effect being detected (Snijders, 2005). Because there were fewer research disciplines (N = 12) and application domains (N = 11) than organisational units (N = 28), lower statistical power may have contributed to the failure to achieve statistically significant effects for the discipline and domain variables.

*#11. What is the share of research productivity in Australia accounted for by the government organizations and not universities? Authors do not provide any context information.*

Although the CSIRO receives a significant proportion of its funding from the Australian Government, it is governed by its own Act and not formally a government organization. As we now explain in the first paragraph of the Method section, the CSIRO shares characteristics in common with both Universities and government organisations. In response to the Reviewer’s feedback, the manuscript now provides statistics on CSIRO’s publication output and patents and the organisation’s role in managing national infrastructure and collections.

# Reviewer #2:

*This paper considers whether organisational units and application domains could influence data sharing practices among researchers. This is clearly a very important issue as data sharing can elevate levels of transparency in scientific endeavours as well as even increase the overall trust and confidence in scientific methods and conclusions. I applaud authors’ efforts to investigate this important question in a unique sample of non-academic researchers.*

Thank you for your positive feedback on our manuscript.

*I must, however, admit that I find it a bit ironic that a manuscript discussing data sharing practices is submitted for publication without openly shared data. I have noted that this is due to ethics application stating that data would not be shared unless it is for validation. Could the data be uploaded to a password protected website with access given to researchers when necessary? I think this could be a good compromise between ethical restrictions and ensuring that data is ready to be shared when needed. If this is not possible, would authors be willing to at least share their R code along with the outputs?*

In line with the informed consent conditions established for the survey we have published the aggregated data for those groups (organisational, disciplinary and domain-specific) where there were ten or more respondents in the data set. In our analyses we included data from groups with seven or more respondents in order to maximise statistical power and the representativeness of our data. The published data therefore do not allow our findings to be replicated. While all of the discipline and domain groups are represented in the published data, only 18 of the 28 organisational units had 10 or more respondents represented in the survey. Furthermore, it is not possible to link the three datasets (which is necessary in order to perform a multilevel analysis) since linkages can only be performed at the individual level of analysis. The published (aggregated) data are available on CSIRO’s Data Access Portal at <https://data.csiro.au/dap/landingpage?pid=csiro:44660>

*Personally, I do not find the paragraph regarding the influence of discipline on data sharing too convincing. Authors argue that data sharing is more common in “big science” disciplines than “small science” disciplines. By default, data would be shared within teams with bigger teams being associated with data being shared to a bigger group of people. It would be helpful if authors could describe at least one study in detail which supports the argument that small science disciplines are less likely to share data than big science disciplines, beyond referring to explanations relating to norms. The analysis conducted by authors do not shed more light on whether this argument is true or not. In this case, perhaps it would be better to focus on how norms within disciplines can influence data sharing practices.*

We have re-written the section describing disciplinary influences on data sharing (commencing on page 4, line 101). We cite two studies (Kim & Stanton, 2016; Kim & Yoon, 2017) which quantify the proportion of variance in research data sharing that is associated with researchers’ disciplines. In addition, we have incorporated Heidorn’s (2008) argument about the economics of data sharing in big science disciplines vs small science disciplines. In all, three factors are identified (ethical, economic and journal/repositories) that contribute to discipline-specific variance in data sharing practices.

*In lines 221-228, intentions to share data, authors report that all items loaded on a single factor, yet no factor analysis is reported in the manuscript. I am somewhat surprised that authors would treat these as a single factor especially there may be different motivations for sharing data within own organisational unit (e.g., collaboration) vs research funders (e.g., requirement) vs general public (e.g., seeing data sharing as important). Alpha of 0.71 may be reflective of this. Have authors considered treating these items as separate outcome variables? Factor analysis should also be reported for contractual and regulatory inhibitors (relating to the domain influences).*

We have removed the statement about the factor analysis results from the Method section and instead provide more detail on the factor analysis in the Results section, including item loadings in the rotated factor solution for all survey items. As Table 2 (page 15) reveals, the seven factor solution has good fit to the data and the communalities for the items assessing intentions to share data are above .50, indicating that they are well defined by the single factor.

*Finally, I cannot comment much on the analytic approach as I have not been trained in multilevel modelling, but could authors report on what sample sizes would be required to reach an adequate power (i.e., 80% or above) in the current analysis? Also, I am not sure why values relating to explained variance in lines 405-408 do not match those in Table 4.*

For testing the effect of a level-one variable, the level-one sample size is of main importance; for testing the effect of a level-two variable it is the level-two sample size (Snijders, 2005). Thus, the power for detecting the organisational unit, discipline and domain components was limited due to having only 28 organisational units, 12 disciplines and 11 domains in the sample. In the revised manuscript we note this issue in the discussion section as a limitation of the study.

To explain how total variance explained is calculated from the figures provided in Table 4, we now illustrate the calculation in the relevant paragraph (see page 22, lines 423 - 428).

Thank you again for your constructive and supportive feedback.

The Authors

References

Heidorn, P. B. (2008). Shedding light on the dark data in the long tail of science. *Library Trends., 57*(2), 280-299. doi:doi:10.1353/lib.0.0036.

Kim, Y., & Stanton, J. M. (2016). Institutional and individual factors affecting scientists' data-sharing behaviors: A multilevel analysis. *Journal of the Association for Information Science and Technology, 67*, 776-799.

Kim, Y., & Yoon, A. (2017). Scientists' data reuse behaviors: A multilevel analysis. *Journal of the Association for Information Science and Technology, 68*(12), 2709-2719.

Snijders, T. (2005). Power and sample size in multilevel linear models. In B. S. Everitt & D. C. Howell (Eds.), *Encyclopedia of Statistics in Behavioral Science.* (Vol. 3, pp. 1570-1573). Chicester: Wiley.

Tenopir, C., Rice, N. M., Allard, S., Baird, L., Borycz, J., Christian, L., . . . Sandusky, R. J. (2020). Data sharing, management, use, and reuse: Practices and perceptions of scientists worldwide. *PLoS ONE, 15*(3). doi:10.1371/journal.pone.0229003

Unal, Y., Chowdhury, G., Kurbanoğlu, S., Boustany, J., & Walton, G. (2019). Research data management and data sharing behaviour of university researchers'. *Information Research: an international electronic journal, 24*(1).
